# Not always towards the null: reconsidering the direction of overadjustment bias

**DOI:** 10.1093/ije/dyag215

**Published:** 2026-10-01

**Authors:** Frerik Smit, Farzaneh Boroumand, Peter W G Tennant, Kylie-Ann Mallitt, Arnaud Chiolero, Anita van Zwieten, Saman Khalatbari-Soltani

**Affiliations:** Population Health Laboratory (#PopHealthLab), University of Fribourg, Fribourg, Switzerland; Swiss School of Public Health (SSPH+), Zurich, Switzerland; School of Public Health, Faculty of Medicine and Health, University of Sydney, Sydney, NSW, Australia; Centre for Kidney Research, Children’s Hospital at Westmead, Westmead, NSW, Australia; Leeds Institute for Data Analytics, University of Leeds, Leeds, United Kingdom; School of Medicine, Faculty of Medicine and Health, University of Leeds, Leeds, United Kingdom; Yale School of Public Health, Yale University, New Haven, CT, United States; School of Public Health, Faculty of Medicine and Health, University of Sydney, Sydney, NSW, Australia; Centre for Kidney Research, Children’s Hospital at Westmead, Westmead, NSW, Australia; School of Clinical Medicine, Faculty of Medicine and Health, University of New South Wales, Sydney, NSW, Australia; Population Health Laboratory (#PopHealthLab), University of Fribourg, Fribourg, Switzerland; Swiss School of Public Health (SSPH+), Zurich, Switzerland; School of Population and Global Health, McGill University, Montreal, QC, Canada; School of Public Health, Faculty of Medicine and Health, University of Sydney, Sydney, NSW, Australia; Centre for Kidney Research, Children’s Hospital at Westmead, Westmead, NSW, Australia; Population Health Laboratory (#PopHealthLab), University of Fribourg, Fribourg, Switzerland; School of Public Health, Faculty of Medicine and Health, University of Sydney, Sydney, NSW, Australia

**Keywords:** bias, overadjustment bias, causal inference, directed acyclic graphs, confounding, collider stratification bias, covariate adjustment, indirect effects

## Abstract

Overadjustment bias occurs when adjusting for a variable increases rather than decreases bias. It commonly arises in studies estimating the total causal effect of an exposure on an outcome due to inappropriate adjustment for a mediator. While such inappropriate mediator adjustment can lead to bias in any direction (towards the null, away from the null, or reverse the direction of effect) even in the absence of other relevant unadjusted variables, this type of overadjustment bias is often described as acting towards the null. Using directed acyclic graphs and a simulated example of adjustment for smoking when estimating the total causal effect of education on the risk of breast cancer, we explain how inappropriately adjusting for a mediator can lead to overadjustment bias in any direction. Correspondingly, we provide general guidance to predict the likely direction of overadjustment bias based on the direction of effect of the relevant paths involved. In general, this bias will be away from the null or in the reverse direction when the mediated pathway being closed by inappropriate adjustment acts in the opposite direction to the combined effect of all remaining pathways between the exposure and the outcome. In contrast, this bias will be towards the null when the effects of these paths act in the same direction.

Key Messages

- Inappropriate adjustment for a mediator when estimating the total causal effect of an exposure on an outcome can introduce bias in any direction (towards the null, away from the null, or reverse the direction of effect); yet, it is commonly asserted that this type of overadjustment bias acts towards the null.
- Overadjustment bias due to the closure of a mediated pathway will act towards the null when the direction of the effect of the mediated pathway is in the same direction as the combined effects of the remaining pathways; the bias will act away from the null or in the reverse direction when these effects are in opposite directions.
- Based on assumptions about the direction of effect of the relevant pathways in the causal structure of a research question of interest, researchers can predict the likely direction of overadjustment bias when a mediator is inappropriately adjusted for.

## Introduction

Overadjustment bias occurs when adjusting for a variable increases rather than decreases bias [1]. It typically arises when data-driven approaches or disciplinary conventions are used to select variables to adjust for to reduce confounding bias when estimating the effect of an exposure on an outcome [1, 2]. For example, in many disciplines, adjusting for variables such as smoking, alcohol, adiposity, and comorbidities is common almost by default. Unfortunately, unless the role of each variable is carefully considered, some variables that are not actually confounders of the relationship between the exposure and the outcome may be adjusted for [1–3]. Rather than reducing bias, adjustment for such variables can increase bias depending on their exact role within a causal structure [1, 2, 4].

The concept of overadjustment bias was first given a formal causal definition by Schisterman *et al.* in 2009 [1]. Since then, *Lu et al*. have classified overadjustment bias into four types, which arise from adjusting for a mediator (type 1), collider (type 2), instrumental variable (type 3), or descendant of the outcome (type 4) [4]. Despite this work, overadjustment bias remains common and underappreciated. This was shown in a review of observational studies of multiple risk factors which found that approximately 7 in 10 included studies mutually adjusted their models for all potential risk factors, which is a method prone to a type of mass overadjustment bias known as table 2 fallacy [5, 6]. Similarly, despite being common in social epidemiology [2], systematic reviews of socioeconomic health inequalities rarely apply strategies to address overadjustment bias [7].

Notably, inappropriate adjustment for colliders (type 2) is generally perceived as the type of overadjustment bias with the most drastic impact. This is largely because adjustment for colliders is known to have the potential to bias effect estimates in any direction, i.e. towards or away from the null and reversals in the direction of effect [2, 8–14]. These implications of collider bias have been exemplified through paradoxical associations such as the birth weight and obesity paradoxes [15, 16]. Correspondingly, many papers exist explaining collider bias and its implications, including its potential direction and magnitude [8–13].

In contrast, inappropriate adjustment for mediators (type 1) is generally perceived as being a less problematic form of overadjustment bias than inappropriate adjustment for colliders (type 2), with only limited discussions of its likely direction. Consequently, the default reference on its direction has been the foundational paper by Schisterman *et al.*, which briefly states that the estimated effect with inappropriate adjustment for a mediator ‘will typically be a null-biased estimate of the total effect’ [1]. Notably, several papers discussing overadjustment bias have since reiterated this expectation [2, 5, 7, 17–21]. While such bias will often be towards the null, the possibility that it can act in other directions (i.e. away from the null or reverse the direction of effect) appears under-recognized. Given the general perception that bias towards the null is less problematic than a bias in other directions, this raises important concerns that researchers may under-appreciate the importance of this type of overadjustment bias.

We therefore aimed to provide a clearer understanding of the direction of overadjustment bias that can arise from inappropriately adjusting for mediators and to illustrate how and when such overadjustment bias might not act towards the null. It is important to note that overadjustment bias can only be evaluated in relation to a specific target estimand [19, 22]. Correspondingly, we focus on estimating the total causal effect (average causal effect) of an exposure on an outcome where mediator adjustment is always inappropriate, rather than direct and indirect effects where adjustment may be appropriate if certain assumptions are met [22]. We begin by explaining the two types of overadjustment bias relevant to mediator adjustment (i.e. types 1 and 2) [4]. We then outline how the direction of type 1 overadjustment bias can be predicted with the aid of a simulated example of estimating the total causal effect of educational attainment level on the risk of breast cancer. We then briefly outline the implications of adjusting for mediators in the presence of other unadjusted relevant variables related to the mediator.

## Understanding overadjustment bias

To explain overadjustment bias, we make use of directed acyclic graphs (DAGs) [3, 23]. For this paper, *E* represents the exposure, *Y* the outcome, and *M* a mediator. Some depicted DAGs also include a collider (*C*), a descendant variable (*D*), and other relevant unmeasured/unadjusted variables (*U*).

Lu *et al*. defined type 1 overadjustment bias, which is our primary focus, as overadjustment bias that arises from inappropriately adjusting for a mediator (also known as an intermediate variable) between the exposure and the outcome [1, 4]. This bias is depicted in Fig. 1. In panel 1a, the total causal effect of the exposure (*E*) on the outcome (*Y*) is shown as acting through two paths: the direct causal path of *E* on *Y* and the mediated causal path of *E* on *Y* passing through the mediator *M.* In panel 1b, *M* has been inappropriately adjusted for (depicted by the box around *M*), leading to the closure of the mediated causal path between *E* and *Y* passing through *M* (i.e. closure of *E* → *M* → *Y*). The result of this inappropriate adjustment is that the association between *E* and *Y* becomes a biased estimate of the total causal effect [1, 2, 4].

**Figure 1:**
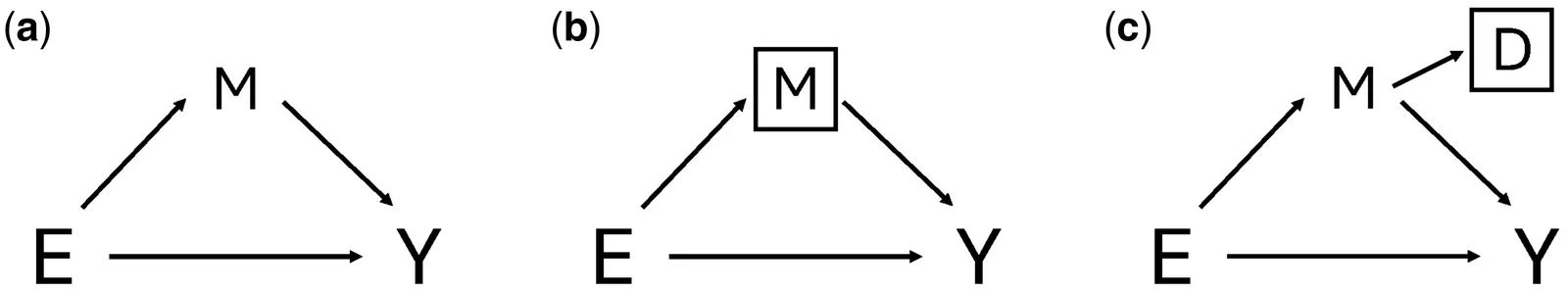
Causal diagrams depicting type 1 overadjustment bias. (a) Scenario without adjustment (obtains an unbiased estimate of the total effect). (b) Scenario with adjustment for a mediator (obtains a biased estimate of the total effect). (c) Scenario with adjustment for a descendant of a mediator (obtains a biased estimate of the total effect). A box around a variable depicts scenarios where that variable is adjusted for (e.g. through covariate adjustment in a regression model, stratification, restriction, or matching).

Type 2 overadjustment bias refers to the bias introduced upon inappropriate adjustment for a collider, defined as the common effect of two or more variables acting through two or more pathways [3, 4, 8, 11]. This bias is depicted in Fig. 2. In panel 2a, the total causal effect of *E* on *Y* is shown as acting through a single direct path (*E* → Y). Notably, in this DAG, both *E* and *Y* also separately cause a collider (*C*); however, this has no bearing on the association between *E* and *Y* because this biasing path (*E* → *C* ← Y) is closed given that *C* has not been adjusted for [3, 4, 8, 11]. In contrast, panel 2b shows a situation in which *C* has been inappropriately adjusted for (depicted by the box around *C*), leading to a biasing path between *E* and *Y* via *C* being opened (i.e. opening of *E* → *C* ← *Y*). This subsequently results in the effect estimate of *E* on *Y* being a biased estimate of the total causal effect [3, 4, 8, 11].

**Figure 2:**
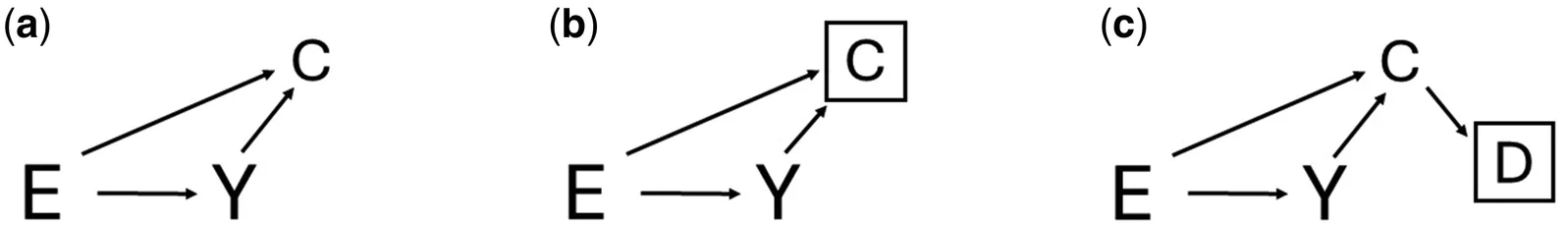
Causal diagrams depicting type 2 overadjustment bias. (a) Scenario without adjustment (obtains an unbiased estimate of the total effect). (b) Scenario with adjustment for a collider (obtains a biased estimate of the total effect). (c) Scenario with adjustment for a descendant of a collider (obtains a biased estimate of the total effect). A box around a variable depicts scenarios where that variable is adjusted for (e.g. through covariate adjustment in a regression model, stratification, restriction, or matching).

Of note, type 1 and type 2 overadjustment bias can occur even without direct adjustment for a mediator or collider, as adjusting for a descendant of a mediator or a descendant of a collider is equivalent to partially adjusting for that variable directly [1, 4]. For example, in Fig. 1c, part of the causal path *E* → *M* → *Y* will be closed by inappropriately adjusting for *D*. Similarly, in Fig. 2c, part of the biasing path *E* → *C* ← *Y* will be opened by inappropriately adjusting for *D*.

## The direction of type 1 overadjustment bias (inappropriate adjustment for a mediator)

Let us now consider the direction and magnitude of overadjustment bias due to the inappropriate adjustment for a mediator. As aforementioned, it is often assumed that type 1 overadjustment bias acts towards the null. However, this bias will only act towards the null in certain situations, whereas in others it can act away from the null or reverse the direction of effect.

Ultimately, the direction and magnitude of type 1 overadjustment bias is determined by the direction and magnitude of the mediated path (*E* → *M* → Y) that is closed by inappropriate mediator adjustment relative to the combined effects of all of the remaining paths between the exposure and the outcome (represented entirely by *E* → *Y* in our simplified example). In cases where the mediated effect and the combined effects of all of the remaining paths are in the same direction (e.g. both have a positive effect), the closure of the mediated pathway through adjusting for the mediator would bias the total effect estimate towards the null. However, when there is a discordance in the direction of effect of the mediated path and the combined effects of all of the remaining paths (e.g. one is positive and the other is negative), adjusting for the mediator would close the mediated path and either lead to an exaggerated estimate of the total effect (i.e. a bias away from the null) or, in scenarios where the mediated effect is stronger than the combined effects of all of the remaining paths, a reversal in the direction of the effect estimate (e.g. where a positive effect switches to becoming negative). In the special case where the entire effect of the exposure on the outcome acted through a single mediator (i.e. there are no other paths between *E* and *Y*), adjusting for that mediator would close the only causal path between the exposure and the outcome, and the biased estimate of the total effect would be exactly null [1].

Under an assumption of no interaction between the exposure and the mediator, these general statements about the direction of type 1 overadjustment bias should hold for effect measures that are collapsible (e.g. risk differences, risk ratios) but not necessarily for non-collapsible effect measures (e.g. odds ratios, hazard ratios) [24]. This is because non-collapsibility produces a mathematical artefact that can result in circumstances where adjustment for a mediator will change the effect estimate beyond the mediator’s influence within the causal structure. In fact, for non-collapsible effect measures, adjustment for a given variable can change effect estimates even in circumstances where said variable is neither a confounder, mediator, nor a collider [24]. In Table 1, we outline how DAGs with signed edges can be used to predict the direction of type 1 overadjustment bias, albeit with some additional assumptions [25–27]. First, to predict the direction of effect of the mediated path based on the expected directions of effect of *E* → *M* and *M* → *Y*, we must assume monotonicity or distributional monotonicity of both effects (i.e. that each of *E* → *M* and *M* → *Y* operate in a consistent direction for all individuals or a distributionally consistent direction within each stratum) [25, 26]. Second, simplifying the combined effects of all the other paths between *E* and *Y* into a singular signed path (i.e. *E* → *Y*) requires either an assumption that all paths from *E* to *Y* apart from *E* → *M* → *Y* have the same sign [26], or that the combined magnitude of the negative signed paths exceeds the combined magnitude of the positive signed paths when specifying a negative sign for E→Y (or vice versa).

**Table 1:** Expected direction of type 1 overadjustment bias under different causal structures.^a^

Signed DAG of causal structure	Target estimand of the total causal effect	Direction of effect of the mediated pathway (*E* → *M* → *Y*)	Direction of the combined effects of the other pathways between *E* and *Y* (*E* → *Y*)	Biased estimate of total effect obtained after overadjusting for the mediator	Expected direction of type 1 overadjustment bias
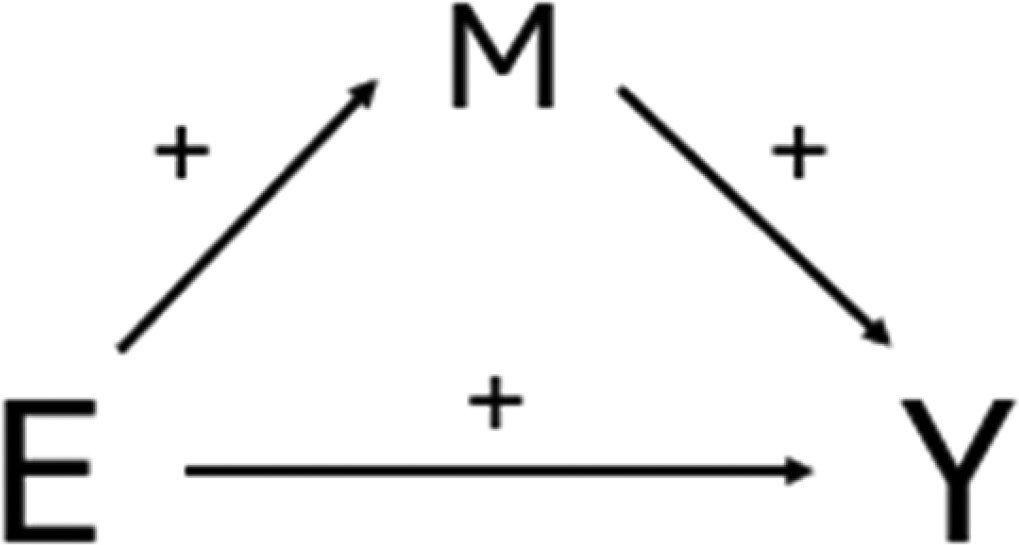	*E* → *Y* + *E* → *M* → Y	Positive	Positive	*E* → *Y*	Downwards and towards the null
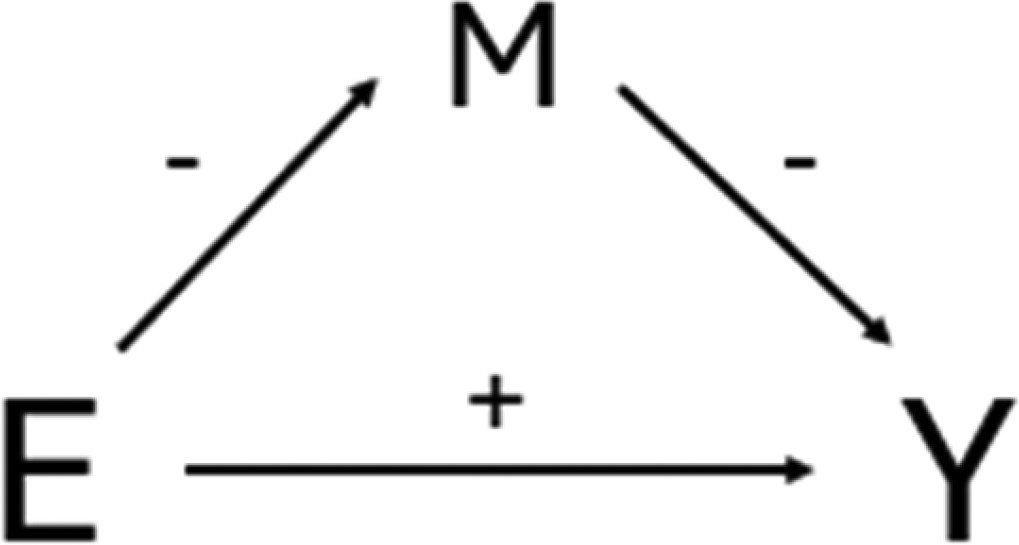	*E* → *Y* + *E* → *M* → *Y*	Positive	Positive	*E* → *Y*	Downwards and towards the null
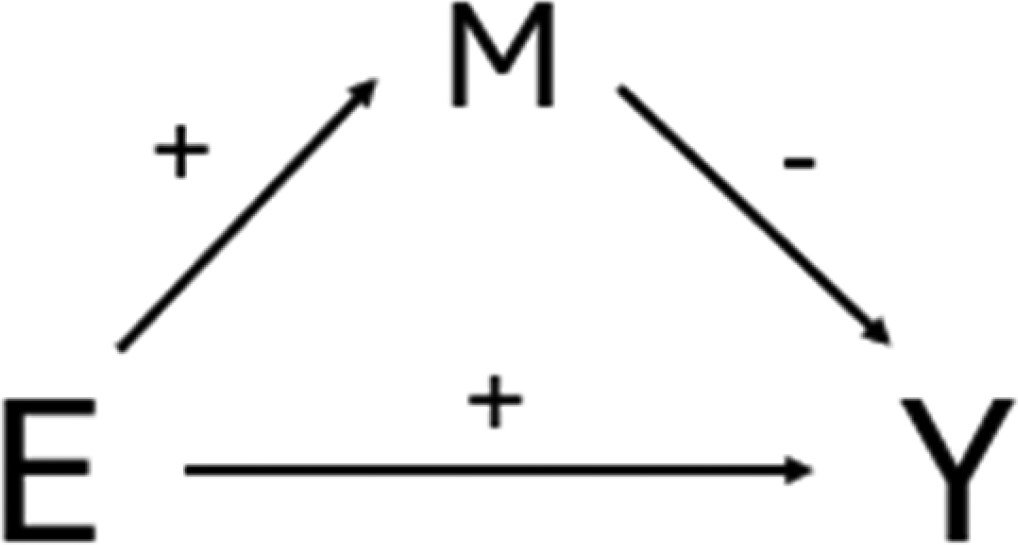	*E* → *Y* + *E* → *M* → Y	Negative	Positive	*E* → Y	Upwards and either away from the null or a reversed direction of effect (when *E* → *M* → *Y* is of a greater relative magnitude than *E* → *Y*)
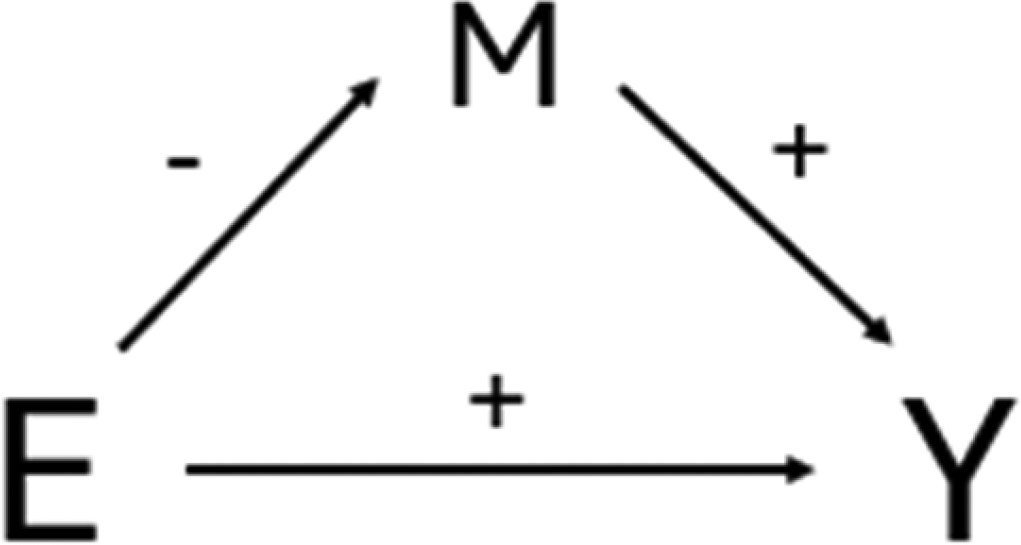	*E* → *Y* + *E* → *M* → *Y*	Negative	Positive	*E* → Y	Upwards and either away from the null or a reversed direction of effect (when *E* → *M* → *Y* is of a greater relative magnitude than *E* → *Y*)
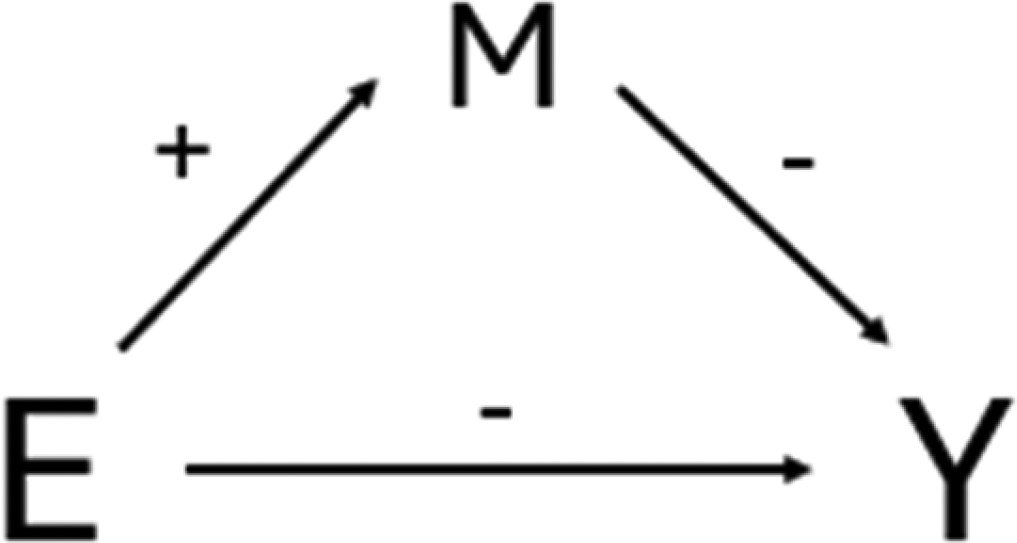	*E* → *Y* + *E* → *M* → Y	Negative	Negative	*E* → *Y*	Upwards and towards the null
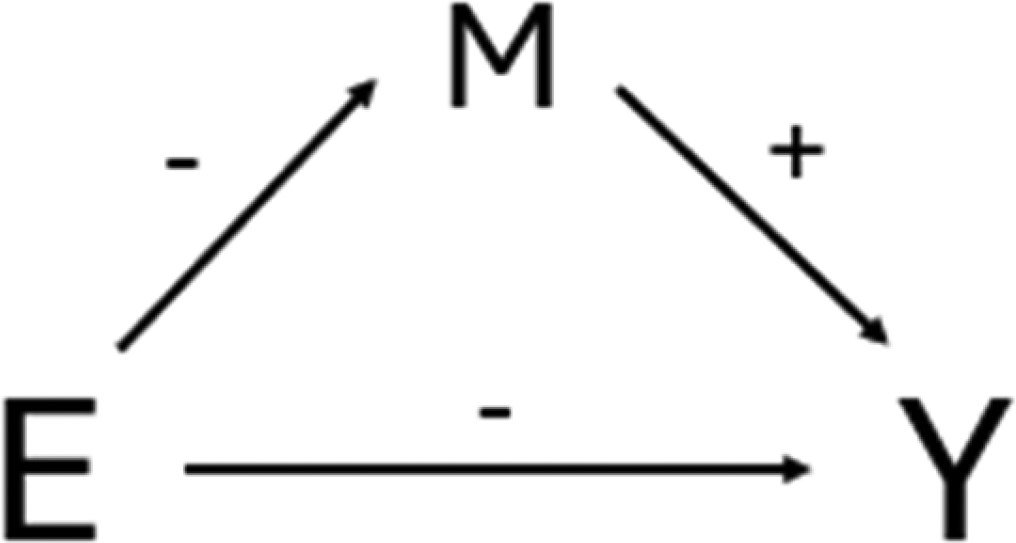	*E* → *Y* + *E* → *M* → *Y*	Negative	Negative	*E* → *Y*	Upwards and towards the null
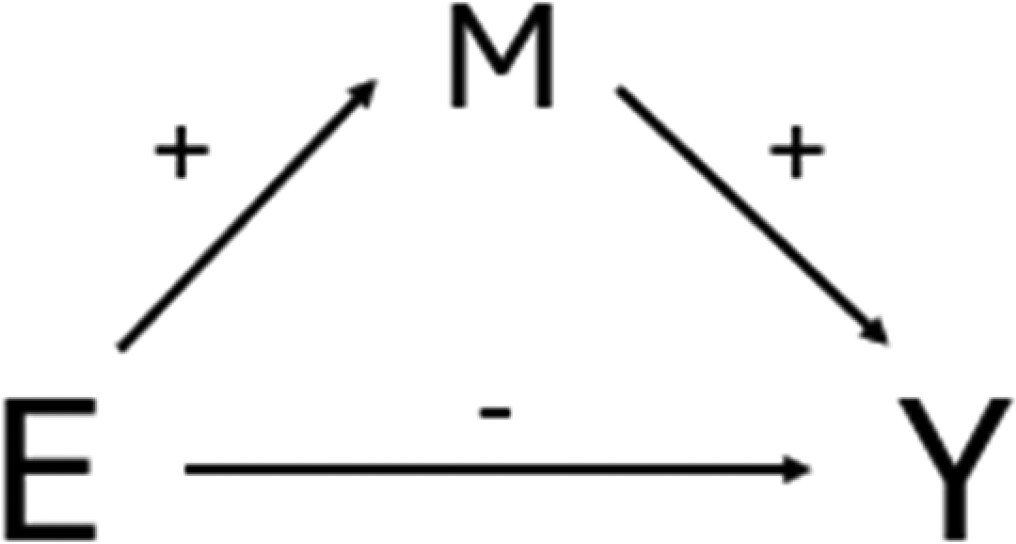	*E* → *Y* + *E* → *M* → *Y*	Positive	Negative	*E* → *Y*	Downwards and either away from the null or a reversed direction of effect (when *E* → *M* → *Y* is of a greater relative magnitude than *E* → *Y*)
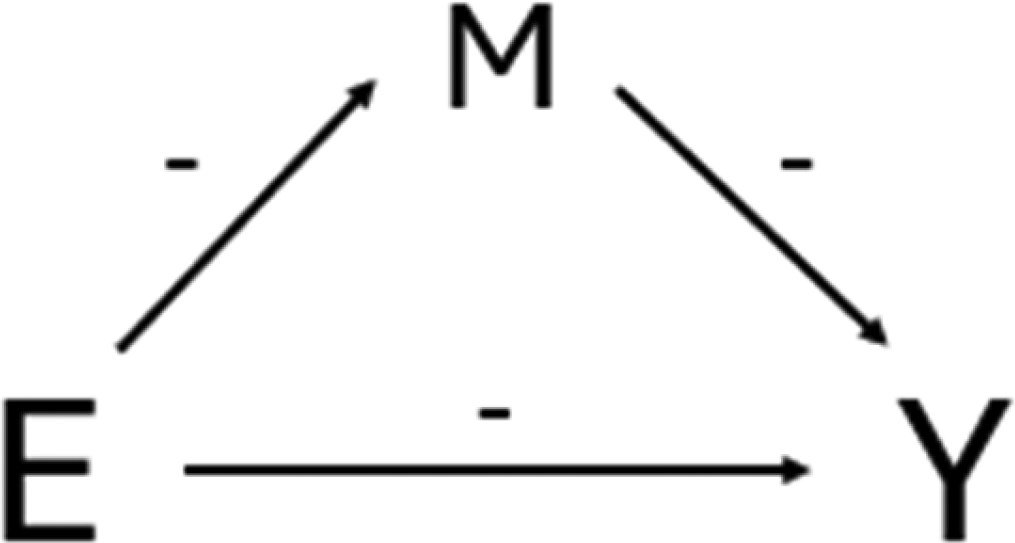	*E* → *Y* + *E* → *M* → *Y*	Positive	Negative	*E* → *Y*	Downwards and either away from the null or a reversed direction of effect (when *E* → *M* → *Y* is of a greater relative magnitude than *E* → *Y*)
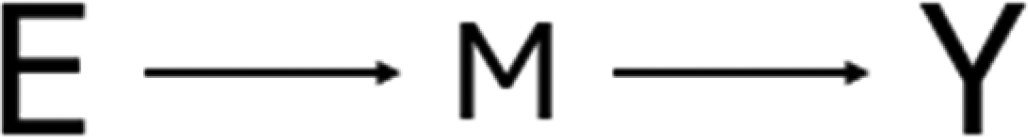	*E* → *M* → *Y*	Positive or negative	Null	Null	To the null exactly.

a *E* → *Y* represents the rest of the total effect (depicted only by one path for educational purposes). *E* → *M* → *Y* corresponds to the product of *E* → *M* and *M* → *Y*.

Alt text: The first column shows directed acyclic graphs of the relationship between an exposure, mediator, and outcome with signs that depict the direction of effect of specific causal edges.

### Illustrative example

Suppose that we are estimating the total causal effect of educational attainment level on the risk of breast cancer among women in Western Europe and that, when doing so, we adjust for smoking by including it as a covariate in a regression model, as this is common practice. We obtain a mean risk difference of 0.13 [95% confidence interval (CI) 0.11–0.14], indicating that women of higher education have a 13 percentage points higher absolute risk of breast cancer (Fig. 3a; Supplementary material). During peer review, it was pointed out that smoking is a mediator between education and breast cancer, meaning we probably introduced overadjustment bias by adjusting for it. In response, we re-ran our analysis without adjustment for smoking, where we obtain a risk difference of 0.02 (95% CI: 0.00–0.03). This suggests that women with higher education may not actually have a substantially higher risk of breast cancer, as the point estimate indicates only a two percentage points higher absolute risk. This thereby illustrates a scenario in which inappropriately adjusting for a mediator introduced type 1 overadjustment bias that strongly biased the estimate of effect away from the null.

**Figure 3:**
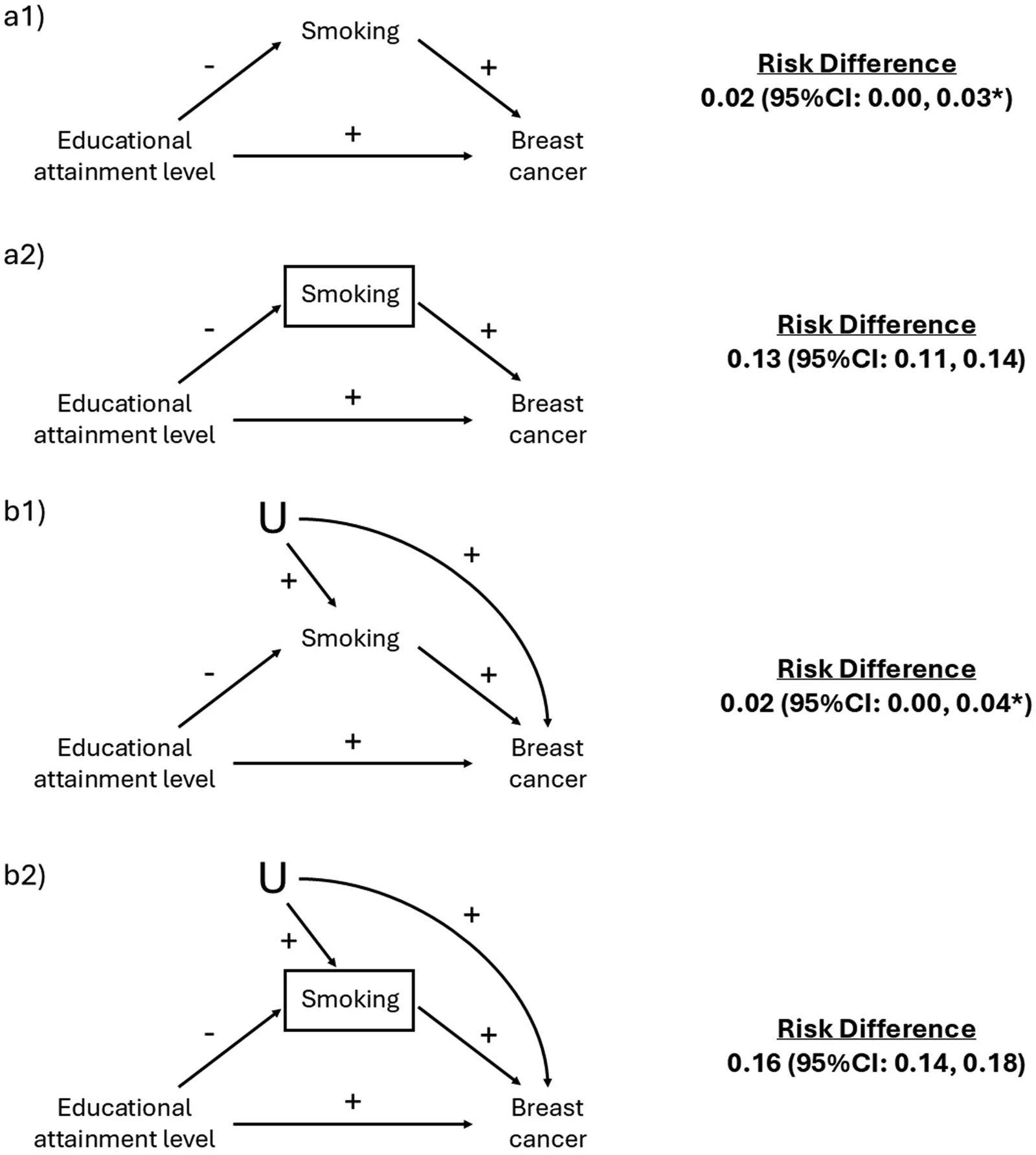
Illustrative example of overadjustment bias due to inappropriate adjustment for the mediator smoking when aiming to estimate the total causal effect of educational attainment level on the risk of breast cancer in women in Western Europe. (a1) Scenario without adjustment for the mediator smoking (obtains an unbiased estimate of the total effect). (a2) Scenario with adjustment for the mediator smoking (obtains a biased estimate of the total effect). (b1) Scenario without adjustment for the mediator smoking in the presence of an unmeasured mediator–outcome confounder (obtains an unbiased estimate of the total effect). (b2) Scenario with adjustment for the mediator smoking in the presence of an unmeasured mediator–outcome confounder (obtains a biased estimate of the total effect). * We conducted two simulation scenarios: one without an unmeasured mediator–outcome confounder (Fig. 3a) and one with an unmeasured mediator–outcome confounder (Fig. 3b). The two scenarios were generated in separate datasets using the same data-generating mechanism, with the only difference being the absence or presence of the unmeasured mediator–outcome confounder (*U*) (see Supplementary material for further details). In turn, small differences in the estimated total effects and corresponding simulation intervals arose due to random variation in the data-generating processes between the two simulations. Specifically, the unadjusted risk difference was 0.02 (95% CI: 0.00–0.03) in the absence of *U* and 0.02 (95% CI: 0.00–0.04) in the presence of *U*, as shown in Fig. 3a and b, respectively. While in the real world, these two scenarios would technically reflect two different causal realities, comparisons between these scenarios are intended to illustrate how the biasing effects from inappropriately adjusting for a mediator vary according to whether an unadjusted mediator–outcome confounder is present in the causal structure. Note, the signs depicted in these DAGs represent the directions of effect specified in our simulated data-generating mechanisms, not assumptions about monotonicity. A box around a variable depicts scenarios where that variable is adjusted for (e.g. through covariate adjustment in a regression model, stratification, restriction, or matching).

Using the information in Table 1, we could have predicted the direction of the type 1 overadjustment bias in this scenario with prior domain knowledge of the direction of effects of the different edges in the causal structure. First, existing literature suggests that higher levels of education reduce the risk of smoking among populations in Western Europe (i.e. the path *E* → *M* is negative) [28, 29]. Second, existing literature suggests that smoking increases the risk of breast cancer (i.e. the path *M* → *Y* is positive) [30, 31]. Taken together, these would indicate that the mediated effect of education on the risk of breast cancer through smoking would be negative (as the negative *E* → *M* multiplied by the positive *M* → *Y* would make *E* → *M* → *Y* negative). Existing literature also suggests that the combined effects of all of the remaining paths between education and breast cancer are likely positive and of a greater relative magnitude than the mediated pathway through smoking [32]. This discordance between the assumed directions of the effect of the mediated pathway and the combined effects of all the remaining pathways based on prior domain knowledge would have suggested that the overadjustment bias introduced by inappropriately adjusting for the mediator smoking would be away from the null, as we observed.

## Implications of adjusting for mediators in the presence of other relevant variables

While we have kept our example simple for illustrative purposes, in practice, researchers should also carefully consider whether there are other relevant variables related to the mediator in their given causal structure—whether measured or unmeasured. This is because their presence may expand the bias implications of inappropriate mediator adjustment beyond just introducing type 1 overadjustment bias. We briefly outline two such instances that researchers are likely to come across.

First, we consider the implications of mediator adjustment in the presence of an unadjusted mediator–outcome confounder (depicted by *U* in Fig. 4a). In this scenario, adjustment for the mediator closes the mediated pathway while also opening a biasing collider path through the mediator–outcome confounder. This occurs because this mediator is also a collider for the exposure and the mediator–outcome confounder, as shown by the paths *E* → *M* and *M* ← *U* → Y colliding at *M* [2, 3, 8]. Adjusting for this mediator therefore introduces a complex double bias, with both type 1 and type 2 overadjustment bias occurring simultaneously. Notably, this bias may be in any direction as both type 1 and type 2 overadjustment bias can introduce bias in any direction. For example, building on the simulated example of estimating the total effect of education on breast cancer, if there was also an unadjusted mediator–outcome confounder (*U*) in this causal structure, then adjustment for smoking could have biased the estimate of the total effect even further away from the null (Fig. 3b; Supplementary material). However, type 1 and type 2 overadjustment biases that occur simultaneously may not act in the same direction, meaning the overall direction of bias will depend on the relative direction and magnitude of both the type 1 and type 2 biases introduced. Readers who are interested in learning more about collider bias are encouraged to refer to explanatory papers [8, 9].

**Figure 4:**
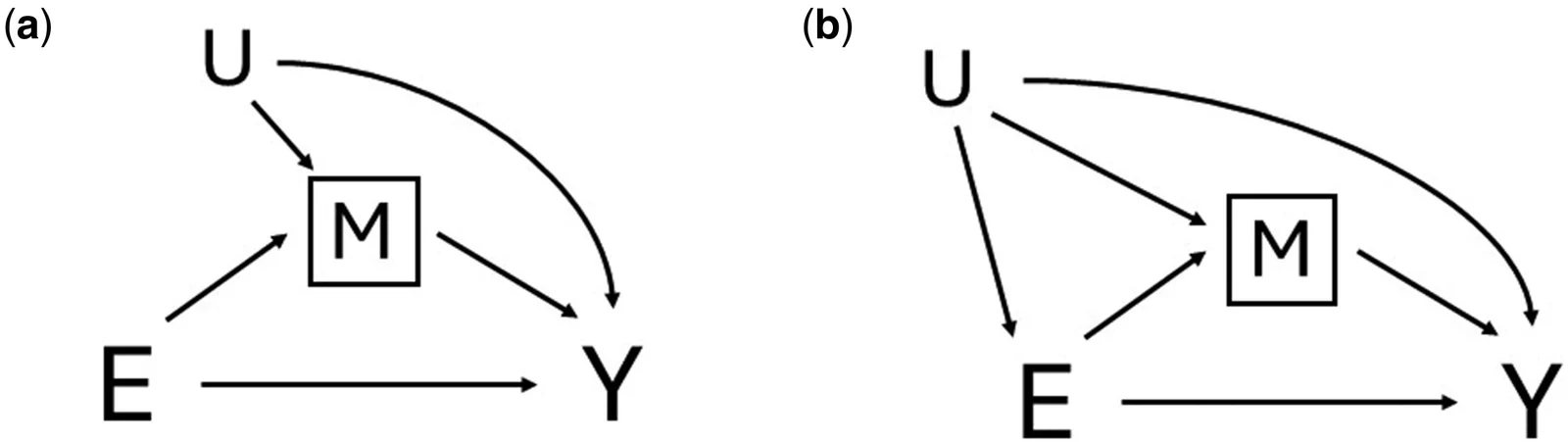
Causal diagrams depicting mediator adjustment in the presence of a relevant unadjusted variable. (a) Mediator–outcome confounder. (b) Exposure-mediator–outcome confounder. A box around a variable depicts scenarios where that variable is adjusted for (e.g. through covariate adjustment in a regression model, stratification, restriction, or matching).

Second, readers should also be aware of the scenario in which an unadjusted variable (depicted by *U* in Fig. 4b) is both a classical confounder for the exposure–outcome relationship and a mediator–outcome confounder [33]. This commonly arises when the exposure and all potential covariates are measured at the same time, leading to an important time-varying confounder not having been measured pre-exposure and, instead, only a post-exposure mediator version of a time-varying confounder being available (depicted by *M* in Fig. 4b) [33]. In theory, adjusting for this mediator could remove the part of the confounding bias from the unmeasured variable that passes through this mediator (i.e. remove *E* ← *U* → *M* → Y). However, while this would reduce some confounding, it would also introduce type 1 and type 2 overadjustment bias. Whether adjustment in this situation is worthwhile therefore depends on whether the residual confounding bias removed is likely of greater relative magnitude than the combined type 1 and type 2 overadjustment bias introduced [4, 33]. We refer readers to Groenwold *et al*. [33] for further details and simulations on this scenario, and we stress the need for extreme caution when considering adjusting for a mediator to reduce residual confounding bias.

## Discussion

Our overview of the implications of inappropriate mediator adjustment when estimating total causal effects aims to support a clearer understanding of the direction of type 1 overadjustment bias. The closure of a mediated pathway from inappropriately adjusting for a mediator will often lead to a bias towards the null—as is generally asserted [2, 5, 7, 17–21]. However, this will only occur when the effect of the mediated pathway being inappropriately closed acts in the same direction as the combined effects of all the remaining pathways between the exposure and the outcome. There will be many instances where this is not the case, as causal effects often act through many different mediated pathways that can have varying directions of effect. To illustrate this, we used a simulated example of estimating the total causal effect of educational attainment level on the risk of breast cancer in which adjusting for a mediator (smoking) led to type 1 overadjustment bias away from the null. Importantly, while this example came from social epidemiology, these issues are relevant to all epidemiological sub-disciplines. For example, in clinical epidemiology, while a drug may have an overall protective effect on a health outcome, it may also have side effects that correspond to adverse mediated pathways.

We have provided an overview of the expected direction of type 1 overadjustment bias in different causal structures (Table 1) that can be used by researchers to predict the direction of this bias for a research question of interest. We also recommend applying this to reviews, where the risk of overadjustment bias and its likely direction could be appraised for included studies as part of critical appraisal [7, 18]. This can be operationalized using the ROBINS-I or ROBINS-E risk of bias assessment tools [7, 18, 34, 35] or by adding items on overadjustment bias to other tools [36].

While we focused on a simple example to illustrate our core point, we acknowledge that causal structures are generally highly complex. There are often other relevant variables that can lead to additional implications of inappropriate mediator adjustment beyond simply introducing type 1 overadjustment bias, most notably mediator–outcome and exposure–mediator–outcome confounders. Nevertheless, even in such complex causal structures, being aware of type 1 overadjustment bias and its likely direction is still important. This is because, even if such variables are adequately adjusted for, inappropriate mediator adjustment may still lead to type 1 overadjustment bias in the direction outlined in Table 1. Alternatively, if such variables are unmeasured or unadjusted, then the overall bias from mediator adjustment will depend on the relative direction and magnitude of all the different biases at play, where the portion of the bias stemming from the closure of the mediated pathway will likely follow the expected directions outlined in Table 1.

There are several simplifications and limitations that we acknowledge. While we focused on overadjustment bias when quantifying total causal effects, issues of overadjustment can also apply to other causal effects of interest (e.g. direct effects) [19]. Relatedly, it is important to remember that overadjustment bias is target estimand specific, such that adjustment for specific mediators would not lead to overadjustment bias when specific estimands such as controlled direct effects are targeted [22]. We also only considered causal effect estimation for single-timepoint exposures using regression-based methods, noting that studies wanting to estimate the joint causal effect of time-varying exposures with multiple timepoints typically require G-methods [37]. We also assumed that other biases such as information and selection bias were absent, that all sufficient causal effect identification assumptions were met, and that there was no effect measure modification or interaction [38, 39]. With regard to our simulations, these were based on a data-generating structure that was intentionally simplified for educational purposes. In a real-world data-generating structure, other variables and biasing paths may influence the impact of inappropriate mediator adjustment.

## Conclusion

Inappropriately adjusting for mediators can bias effect estimates in any direction (i.e. towards the null, away from the null, and reversed direction of effect) even in the absence of other relevant unadjusted variables. The bias introduced through inappropriately adjusting for a mediator (type 1 overadjustment bias) can therefore potentially be as important as bias from inappropriately adjusting for a collider (type 2 overadjustment bias). These implications highlight the need for researchers wishing to estimate causal effects to be cautious in their selection of variables to adjust for, with a need for particular attention to overadjustment bias which has historically been underappreciated.

## Ethics approval

This study did not require ethics approval as it did not include any human participants.

## Supplementary Material

dyag215_Supplementary_Data

## Data Availability

The simulations can be reproduced using the R code provided in the supplementary material.
