# Supplementary material for: Not always towards the null: reconsidering the direction of overadjustment bias
Source: Int J Epidemiol. 2026 Oct 1;55(5):dyag215. doi: 10.1093/ije/dyag215 (PMC13629551; doi:10.1093/ije/dyag215)
Supplement: Supplementary file 1 [file dyag215_supplementary_data.docx]

**Supplementary Material**

**Study Title:** Not always towards the null: reconsidering the direction of overadjustment bias

**Table of Contents**

[Description of Scenario 1 Simulation (Type 1 Overadjustment Bias)	2](#_Toc222734502)

[Description of Scenario 2 Simulation (Simultaneous Type 1 and Type 2 Overadjustment Bias)	2](#_Toc222734503)

[Simulation Methods	2](#_Toc222734504)

[Section 1: Demonstration in One Simulated Dataset	3](#_Toc222734505)

[1.0: Simulating the whole population:	4](#_Toc222734506)

[1.1: Total effect of education on breast cancer using GLM without adjusting for the mediator smoking:	5](#_Toc222734507)

[1.2: Total effect of education on breast cancer using mediation analysis with adjustment for the mediator smoking:	6](#_Toc222734508)

[1.3: Biased model where the mediator smoking is inappropriately adjusted for	7](#_Toc222734509)

[1.4: Comparison:	8](#_Toc222734510)

[Section 2: Description of Monte Carlo Simulations for Scenario 1 (Type 1 Overadjustment Bias)	9](#_Toc222734511)

[Section 3: Description of Monte Carlo Simulations for Scenario 2 (Simultaneous Type 1 and Type 2 Overadjustment Bias)	11](#_Toc222734512)

# Description of Scenario 1 Simulation (Type 1 Overadjustment Bias)

This simulation demonstrates type 1 overadjustment bias, where the indirect effect through the mediator (E→M→Y) and the direct effect of the exposure (E→Y) operate in opposite directions.

Indirect effect (ACME): Negative (education reduces smoking, thereby reducing the risk of breast cancer).

Direct effect (ADE): Positive (education directly increases the risk of breast cancer).

Total effect: Positive because the direct effect is greater in magnitude than the indirect effect.

A key insight from this scenario is that type 1 overadjustment bias does not always act towards the null. Instead, the direction of bias depends on the relative magnitudes of the direct and indirect effects.

# Description of Scenario 2 Simulation (Simultaneous Type 1 and Type 2 Overadjustment Bias)

This simulation demonstrates type 1 and type 2 overadjustment bias occurring simultaneously. As was the case in the above scenario, this scenario also has the indirect effect through the mediator (E→M→Y) and the direct effect of the exposure (E→Y) operating in opposite directions. In addition, this scenario also contains an unmeasured mediator outcome confounder (U).

# Simulation Methods

We conducted a simulation study in which the exposure (education) had a negative indirect effect on breast cancer through the mediator smoking but a positive direct effect, where the direct effect was larger in magnitude than the indirect effect. Candidate coefficients for the data-generating model were identified using a grid search, where we aimed to have a moderate difference between the true total effect and the biased estimate. Based on the selected parameters, we generated a population of 10,000,000 observations and then drew a random sample of 10,000 individuals with replacement. From this sample, we estimated the total effect of education on breast cancer using three approaches: (i) a correctly specified logistic regression model that does not adjust for the mediator smoking, (ii) causal mediation analysis, and (iii) a logistic regression model that inappropriately adjusts for the mediator. In a second step, to demonstrate that the results obtained in Step 1 were not due to chance in a single random sample, we repeated Step 1 across 10,000 replications using a Monte Carlo simulation to estimate the mean risk difference and corresponding confidence intervals under each approach. We conduct this simulation for both scenario 1 and scenario 2, where for scenario 2 we also add an unmeasured mediator-outcome confounder (U) which has a positive effect on both the mediator and the outcome.

We note that, to reduce the necessary computing power or time to run these simulations, it is possible to reduce the number of replications performed. This can be achieved by specifying a lower number of “sims” within the “mediate()” functions and/or reducing the number of replications within the first argument of the “replicate()” functions. Alterations of simulations might lead to minor changes in the results, as an artefact of simulations.

# Section 1: Demonstration in One Simulated Dataset

The total effect (risk difference) is approximately 0.02 (RD_glm and med$tau.coef ), as estimated by both the simple generalised linear model (GLM) and the mediation analysis.

The biased model, in which the mediator smoking is inappropriately adjusted for, yields a larger effect of approximately 0.13 (RD_overadjusted). This pattern can be clearly seen in the results:

- **GLM without mediator adjustment:** The total effect of education on breast cancer (risk difference) is 0.02 (RD_glm).
- **Proper mediation analysis:** The total effect of education on breast cancer (risk difference) is also 0.02 (med$tau.coef).
- **Biased model (inappropriately adjusting for smoking):** The estimated effect of education on breast cancer increases to 0.13 (RD_overadjusted).

These results demonstrate that type 1 overadjustment bias does not always lead to a bias towards the null. Instead, in situations where the indirect effect through the mediator (E→M→Y) and the direct effect of the exposure (E→Y) operate in opposite directions, inappropriately adjusting for the mediator can lead to a bias away from the null or reverse the direction of effect. In this scenario, given that the direct effect was greater in magnitude than the indirect effect, the bias acted away from the null. However, if the indirect effect had been larger in magnitude than the direct effect, inappropriately adjusting for the mediator would have led to a reverse in the direction of effect.

## 1.0: Simulating the whole population:

```
set.seed(1368)
n <- 1e7
education <- rbinom(n, 1, 0.5)

# make education's protective effect on smoking weaker
p_smoking <- ifelse(education == 1, 0.2, 0.6)
smoking <- rbinom(n, 1, p_smoking)

# Stronger direct effect of education on cancer
log_odds_cancer <- -2 + 0.7 * education + 1.4 * smoking
p_cancer <- plogis(log_odds_cancer)
breast_cancer <- rbinom(n, 1, p_cancer)

data <- data.frame(education, smoking, breast_cancer)

# Total effect
model_total <- glm(breast_cancer ~ education, data = data, family = binomial)
#summary(model_total)

#---true effects of the whole population
true_total_effect<-model_total$coefficients[2]
p0 <- mean(predict(model_total,
                   newdata = data.frame(education=0),
                   type="response"))

# Predictions under education=1
p1 <- mean(predict(model_total,
                   newdata = data.frame(education=1),
                   type="response"))

# Risk difference from glm
RD_True_glm <- p1 - p0
```

## 1.1: Total effect of education on breast cancer using GLM without adjusting for the mediator smoking:

```
#--------------------- sample data
set.seed(1368)
sample_data <- data[sample(nrow(data), 10000, replace = TRUE), ]


# Total effect
print("Total effect from a model not adjusted for smoking")
sample_model_total <- glm(breast_cancer ~ education, data = sample_data, family = binomial)
summary(sample_model_total)


# Predictions under education=0
p0 <- mean(predict(sample_model_total,
                   newdata = data.frame(education=0),
                   type="response"))

# Predictions under education=1
p1 <- mean(predict(sample_model_total,
                   newdata = data.frame(education=1),
                   type="response"))

# Risk difference from glm
RD_glm <- p1 - p0

print("RD_glm")
RD_glm
```

## 1.2: Total effect of education on breast cancer using mediation analysis with adjustment for the mediator smoking:

```
#total effect using mediation analysis
set.seed(1368)

library(mediation)
mediator_model <- glm(smoking ~ education, data = sample_data, family = binomial)

outcome_model <- glm(breast_cancer ~ education + smoking, data = sample_data, family = binomial)
med <- mediate(mediator_model, outcome_model,
               treat = "education",
               mediator = "smoking",
               robustSE = TRUE,
               sims = 1000)

summary(med)
print("med$tau.coef")
med$tau.coef
```

## 1.3: Biased model where the mediator smoking is inappropriately adjusted for

```
#-overadjusted model

print("overadjusted model")

overadjusted_model <- glm(breast_cancer ~ education + smoking, data = sample_data, family = binomial)
summary(overadjusted_model)


newdata0 <- sample_data
newdata0$education <- 0

newdata1 <- sample_data
newdata1$education <- 1

# Predicted probabilities under each scenario
p0 <- mean(predict(overadjusted_model, newdata=newdata0, type="response"))
p1 <- mean(predict(overadjusted_model, newdata=newdata1, type="response"))

# Risk difference (education=1 vs 0)
RD_overadjusted <- p1 - p0
RD_overadjusted
```

## 1.4: Comparison:

```
#total effect of educ on cancer by not including mediator
RD_glm

#total effect of educ on cancer by including mediator in a proper mediation analysis
med$tau.coef

#effect of education on cancer by overadjustion for mediator
RD_overadjusted
```

# Section 2: Description of Monte Carlo Simulations for Scenario 1 (Type 1 Overadjustment Bias)

To assess the stability of the findings, we repeated the simulation using a Monte Carlo approach with 10,000 replications. Across replications, the average estimates and their confidence intervals closely matched those obtained from the single simulated dataset.

- **GLM without mediator:** The mean risk difference was 0.02 (95% CI: 0.00 to 0.03).
- **Mediation analysis:** The mean risk difference was 0.02 (95% CI: 0.00 to 0.03).
- **Biased model (inappropriately adjusting for smoking):** The mean risk difference was 0.13 (95% CI: 0.11 to 0.14).

These results indicate that the earlier observation is unlikely to be due to chance in a single sample. The total effect estimates from the GLM and mediation analysis align closely, while the biased model again produces a larger estimate that is thereby biased away from the null.

```
#----------------------------- Monte carlo simulation
library(mediation)
library(dplyr)


#--- Generate the population

set.seed(1368)
n <- 1e7
education <- rbinom(n, 1, 0.5)

# make education's protective effect on smoking weaker
p_smoking <- ifelse(education == 1, 0.2, 0.6)
smoking <- rbinom(n, 1, p_smoking)

# Stronger direct effect of education on cancer
log_odds_cancer <- -2 + 0.7 * education + 1.4 * smoking
p_cancer <- plogis(log_odds_cancer)
breast_cancer <- rbinom(n, 1, p_cancer)

population <- data.frame(education, smoking, breast_cancer)

#--- Function to compute effects from one sample
one_run <- function(population, sample_size = 10000) {
  sample_data <- population[sample(nrow(population), sample_size, replace = TRUE), ]

  # Total effect: glm without mediator
  sample_model_total <- glm(breast_cancer ~ education, data = sample_data, family = binomial)
  p0 <- mean(predict(sample_model_total, newdata = data.frame(education=0), type="response"))
  p1 <- mean(predict(sample_model_total, newdata = data.frame(education=1), type="response"))
  RD_glm <- p1 - p0

  # Mediation analysis
  mediator_model <- glm(smoking ~ education, data = sample_data, family = binomial)
  outcome_model  <- glm(breast_cancer ~ education + smoking, data = sample_data, family = binomial)
  med <- mediate(mediator_model, outcome_model,
                 treat = "education", mediator = "smoking",
                 robustSE = TRUE, sims = 100)
  RD_med <- med$tau.coef

  # Overadjusted model
  over_model <- glm(breast_cancer ~ education + smoking, data = sample_data, family = binomial)
  newdata0 <- sample_data; newdata0$education <- 0
  newdata1 <- sample_data; newdata1$education <- 1
  p0_over <- mean(predict(over_model, newdata=newdata0, type="response"))
  p1_over <- mean(predict(over_model, newdata=newdata1, type="response"))
  RD_over <- p1_over - p0_over

  c(RD_glm = RD_glm, RD_med = RD_med, RD_overadjusted = RD_over)
}

#--- Repeat 10000 times
res <- replicate(10000, one_run(population, sample_size = 10000))
res <- t(res) %>% as.data.frame()

#--- Summarise results
summary_results <- res %>%
  summarise(
    mean_RD_glm = mean(RD_glm),
    se_RD_glm   = sd(RD_glm),
    ci_low_RD_glm = mean_RD_glm - 1.96*se_RD_glm,
    ci_up_RD_glm  = mean_RD_glm + 1.96*se_RD_glm,

    mean_RD_med = mean(RD_med),
    se_RD_med   = sd(RD_med),
    ci_low_RD_med = mean_RD_med - 1.96*se_RD_med,
    ci_up_RD_med  = mean_RD_med + 1.96*se_RD_med,

    mean_RD_over = mean(RD_overadjusted),
    se_RD_over   = sd(RD_overadjusted),
    ci_low_RD_over = mean_RD_over - 1.96*se_RD_over,
    ci_up_RD_over  = mean_RD_over + 1.96*se_RD_over
  )

print(round(summary_results,2))
```

# Section 3: Description of Monte Carlo Simulations for Scenario 2 (Simultaneous Type 1 and Type 2 Overadjustment Bias)

To evaluate how overadjustment bias due to the inappropriate adjustment of the mediator smoking is impacted by the additional presence of an unmeasured mediator-outcome confounder (U), we undertake similar steps as for Scenario 1, with Monte Carlo simulations with 10,000 replications. Across replications, the average estimates of the models without adjustment for the mediator smoking and their confidence intervals closely matched those obtained from the Monte Carlo simulations from Scenario 1. Meanwhile, for the model in which the mediator smoking was inappropriately adjusted for, the average risk difference was higher than the corresponding average risk difference observed in Scenario 1.

- **GLM without mediator:** The mean risk difference was 0.02 (95% CI: 0.00 to 0.04).
- **Mediation analysis:** The mean risk difference was 0.02 (95% CI: 0.00 to 0.04).
- **Biased model (inappropriately adjusting for smoking):** The mean risk difference was 0.16 (95% CI: 0.14 to 0.18).

Therefore, we observe that the overadjustment bias resulting from the inappropriate adjustment for the mediator smoking was even further away from the null in this scenario with the presence of an unmeasured mediator-outcome confounder than it was in Scenario 1 where this unmeasured mediator-outcome confounder was absent.

```
#----------------------------- Monte carlo simulation
library(mediation)
library(dplyr)

set.seed(1368)
n <- 1e7

# Exposure
education <- rbinom(n, 1, 0.5)

# ---- Add mediator–outcome confounder (U) ----
# U
U <- rnorm(n, mean = 1, sd = 0.5)

# ---- Mediator model (smoking) ----
# Smoking depends on education (protective effect) and U (Positive effect)
logit_p_smoking <- qlogis(ifelse(education == 1, 0.2, 0.6)) + 0.8 * U
p_smoking <- plogis(logit_p_smoking)
smoking <- rbinom(n, 1, p_smoking)

# ---- Outcome model (breast cancer) ----
# Cancer depends on education, smoking, and U (Positive effect)
log_odds_cancer <- -2 + 0.7 * education + 1.4 * smoking + 0.8 * U
p_cancer <- plogis(log_odds_cancer)
breast_cancer <- rbinom(n, 1, p_cancer)

# ---- Combine data ----
population <- data.frame(education, smoking, breast_cancer)

#--- Function to compute effects from one sample
one_run <- function(population, sample_size = 10000) {
  sample_data <- population[sample(nrow(population), sample_size, replace = TRUE), ]

  # Total effect: glm without mediator
  sample_model_total <- glm(breast_cancer ~ education, data = sample_data, family = binomial)
  p0 <- mean(predict(sample_model_total, newdata = data.frame(education=0), type="response"))
  p1 <- mean(predict(sample_model_total, newdata = data.frame(education=1), type="response"))
  RD_glm <- p1 - p0

  # Mediation analysis
  mediator_model <- glm(smoking ~ education, data = sample_data, family = binomial)
  outcome_model  <- glm(breast_cancer ~ education + smoking, data = sample_data, family = binomial)
  med <- mediate(mediator_model, outcome_model,
                 treat = "education", mediator = "smoking",
                 robustSE = TRUE, sims = 100)
  RD_med <- med$tau.coef

  # Overadjusted model
  over_model <- glm(breast_cancer ~ education + smoking, data = sample_data, family = binomial)
  newdata0 <- sample_data; newdata0$education <- 0
  newdata1 <- sample_data; newdata1$education <- 1
  p0_over <- mean(predict(over_model, newdata=newdata0, type="response"))
  p1_over <- mean(predict(over_model, newdata=newdata1, type="response"))
  RD_over <- p1_over - p0_over

  c(RD_glm = RD_glm, RD_med = RD_med, RD_overadjusted = RD_over)
}

#--- Repeat 10,000 times
res <- replicate(10000, one_run(population, sample_size = 10000))
res <- t(res) %>% as.data.frame()

#--- Summarise results
summary_results <- res %>%
  summarise(
    mean_RD_glm = mean(RD_glm),
    se_RD_glm   = sd(RD_glm),
    ci_low_RD_glm = mean_RD_glm - 1.96*se_RD_glm,
    ci_up_RD_glm  = mean_RD_glm + 1.96*se_RD_glm,

    mean_RD_med = mean(RD_med),
    se_RD_med   = sd(RD_med),
    ci_low_RD_med = mean_RD_med - 1.96*se_RD_med,
    ci_up_RD_med  = mean_RD_med + 1.96*se_RD_med,

    mean_RD_over = mean(RD_overadjusted),
    se_RD_over   = sd(RD_overadjusted),
    ci_low_RD_over = mean_RD_over - 1.96*se_RD_over,
    ci_up_RD_over  = mean_RD_over + 1.96*se_RD_over
  )
print(round(summary_results,2))
```
